# Supplementary material for: Group membership biases children’s evaluation of evidence
Source: Nat Commun. 2025 Dec 15;16:11245. doi: 10.1038/s41467-025-66085-0 (PMC12717042; doi:10.1038/s41467-025-66085-0)
Supplement: Supplementary file 1 — Supplementary Information [file 41467_2025_66085_MOESM1_ESM.pdf]

Supplementary Information

Supplementary Methods

Study 1: Description of the statistical analysis

To analyze how group membership influenced children's decision to evaluate evidence through opening the boxes, we fitted a linear model to predict the number of boxes opened (1-10), by condition (No Group, Group) and age (continuous, 4-6), and their interaction as fixed effects.

Full Model for Number of Boxes	Boxes (1-10) ~ Condition (No Group / Group Condition) * Age (4-6, continuous)

We next checked for the assumption of the absence of collinearity. Generalized variance inflation factors (vif) were derived using the vif function of the R package car (version 3.1.3)[1] applied to the reduced model containing only the main effects. This suggested that collinearity was not an issue. We then checked the model residuals visually, which fulfilled the assumption to be normally distributed. There were no influential cases. The homoscedasticity assumption was assessed using both the Breusch–Pagan test and visual inspection of the residuals versus fitted values plot. The Breusch–Pagan test was non-significant (p = .49), suggesting no statistical evidence of heteroscedasticity. However, the residuals plot suggested a pattern of non-constant variance, with increasing spread at higher fitted values. Given this discrepancy between the formal test and the visual diagnostics, we ensured the validity of our results with an additional Generalized Linear Mixed Model (GLMM) reported below. For effect sizes, we report the standard coefficient of determination (R^2) for the linear models.

To avoid an increased type 1 error risk due to multiple testing, we then tested the overall effect of the predictors. Therefore, we compared the model fit of the full model to a null model including only the intercept. Then, to determine the effects of each predictor, we compared the full model to reduced models lacking the predictor of interest.

The full model for boxes containing condition, age, and their interaction as predictors was a significantly better fit than the null model ($\chi^2(3)$ = 131.93, p = .012, R^2 = 0.13). Next, we compared the full model to reduced models. There was a significant effect of condition ($\chi^2(1)$ = 78.15, p = .012, R^2 = 0.08), such that how many boxes children opened (1-10) depended on whether they were in the Group Condition or No Group Condition. On average, children in the Group

Condition opened about four boxes while children in the No Group Condition opened about six boxes. There was no statistically significant interaction between condition and age ($\chi^2(1) = 38.10$, $p = .070$, $R^2 = 0.04$), nor was there a statistically significant effect of age ($\chi^2(1) = 37.94$, $p = .076$, $R^2 = 0.04$).

Table S1. **Full model (lm) output for number of boxes in Study 1**. Estimates, standard errors (SE), and likelihood ratio test output for the single effects. The factor condition was dummy coded with the No Group Condition as the reference level. Confidence intervals (CI) were derived using the confint function. Likelihood ratio tests used χ^2 statistics and were two-sided. To account for multiple comparisons, we first conducted a full-null model comparison before testing individual effects.

	estimates	SE	CI_{Lower}	CI_{Upper}	χ^2	*df*	*p*
(Intercept)	16.94	4.45	8.07	25.82			
Condition	-12.09	5.67	-23.38	-0.80	78.15	1	.012
Age	-1.94	0.77	-3.47	-0.40	37.94	1	.076
Condition: Age	1.80	1.01	-0.21	3.81	38.10	1	.070

As pre-registered, we also fitted a generalized linear mixed model (GLMM) [2] to predict the number of boxes children opened by condition. In this model, opening each box was treated as a binary response variable (yes/no), with each participant having the possibility of opening 10 total boxes. We included the predictors condition, age, and their interaction as fixed effects, and a random intercept for individual identity. Collinearity was not an issue and there was an absence of influential cases. Effect sizes of GLMMs and the associated fixed effects were calculated as the semi-partial R^2 using the partR2 package (version 0.9.2)[3].

As in the analysis above, the full model for number of boxes was a significantly better fit than the null model ($\chi^2(3) = 11.24$, $p = .011$, $R^2 = 0.16$). We then compared a reduced model without the interaction term to models without a predictor of interest. We found a significant effect of condition ($\chi^2(1) = 5.64$, $p = .018$, $R^2 = 0.05$), such that how many boxes children opened varied across the No Group and Group Conditions. There was no statistically significant effect of age ($\chi^2(1) = 3.53$, $p = .060$, $R^2 = 0.02$). Lastly, we compared the reduced model without the interaction term to the full model. There was a significant interaction between condition and age ($\chi^2(1) = 4.02$, $p = .045$, $R^2 = 0.03$).

Table S2. **Full model (GLMM) output for number of boxes in Study 1**. Estimates, standard errors (SE), and likelihood ratio test output for the single effects. The factor condition was dummy coded with the No Group Condition as the reference level. Confidence intervals were derived using the 1000 parametric bootstraps. Likelihood ratio tests

used χ^2 statistics and were two-sided. To account for multiple comparisons, we first conducted a full-null model comparison before testing individual effects.

	estimates	SE	CI_{Lower}	CI_{Upper}	χ^2	*df*	*p*
(Intercept)	9.71	3.32	3.56	17.17			
Condition	-9.43	4.08	-18.09	-1.49	78.15	1	.018
Age	-1.53	0.57	-2.78	-0.49	37.94	1	.060
Condition: Age	1.43	0.72	0.00	2.98	38.10	1	.045

For our second dependent variable (belief formed), as pre-registered, we fitted a generalized linear model, as belief was a binary response variable (0 = incorrect belief, 1 = correct belief). We included the predictors condition (Group, No Group), age (4-6), and their interaction as fixed effects. Next, we checked the model assumptions. Collinearity was not an issue (largest vif = 1.22) and there was an absence of influential cases. Effect sizes were calculated as the Nagelkerke R^2 using the rsq package[4] (version 2.7).

Full Model for Belief about Boxes	Belief (correct belief/incorrect belief) ~ Condition (No Group / Group Condition) * Age (4-6, continuous)

The full model for belief was a significantly better fit than the null model ($\chi^2(3) = 13.24$, $p = .004$, $R^2 = 0.21$), and once again, there was a significant effect of condition ($\chi^2(1) = 8.53$, $p < .003$, $R^2 = 0.14$) such that whether children believed there were more elephants or lions in the boxes depended on whether they were in the Group Condition or No Group Condition. There was not a statistically significant interaction between condition and age ($\chi^2(1) = 3.03$, $p = .082$, $R^2 = 0.05$), though there was a significant effect of age ($\chi^2(1) = 4.55$, $p = .033$, $R^2 = 0.08$), such that older children more often held the incorrect belief that there were more elephants in the boxes.

Table S3. **Full model (glm) output for belief about the boxes in Study 1**. Estimates, standard errors (SE), and likelihood ratio test output for the single effects. The factor condition was dummy coded with the No Group Condition as the reference level. Confidence intervals were derived using the confint function. Likelihood ratio tests used χ^2 statistics and were two-sided. To account for multiple comparisons, we first conducted a full-null model comparison before testing individual effects.

	estimates	SE	CI_{Lower}	CI_{Upper}	χ^2	*df*	*p*
(Intercept)	7.88	3.20	2.12	14.91			

Condition	-8.07	3.94	-16.33	-0.63	8.53	1	.003
Age	-1.35	0.55	-2.55	-0.36	4.55	1	.033
Condition: Age	1.19	0.70	-0.15	2.63	3.03	1	.082

To investigate whether the effect of condition on belief was mediated by the number of boxes opened, we conducted an exploratory mediation analysis. Condition (Group, No Group) was treated as the intervention variable, the number of boxes opened as the mediator, and the belief formed as the outcome variable. This analysis was carried out using the mediation package[5] (version 4.5.1). This analysis revealed that the group manipulation effect on the outcome was significantly mediated through the number of boxes that children opened, as indicated by the significant average mediation effect (ACME = -0.12, 95% CI [-0.24, -0.01], p = .030). 47.1% of the total effect was mediated by the number of boxes opened, as indicated by the significant average proportion mediated (Estimate = 0.47, 95% CI [0.01, 1.74], p = .049). The total effect of the condition (including direct and indirect effect) on belief formed was significant (Estimate = -0.25, 95% CI [-0.46, -0.03], p = .027), while the direct effect (ADE, c') of condition on belief alone was not significant once the mediation variable was considered (ADE = -0.13, 95% CI [-0.33, 0.06], p = .186).

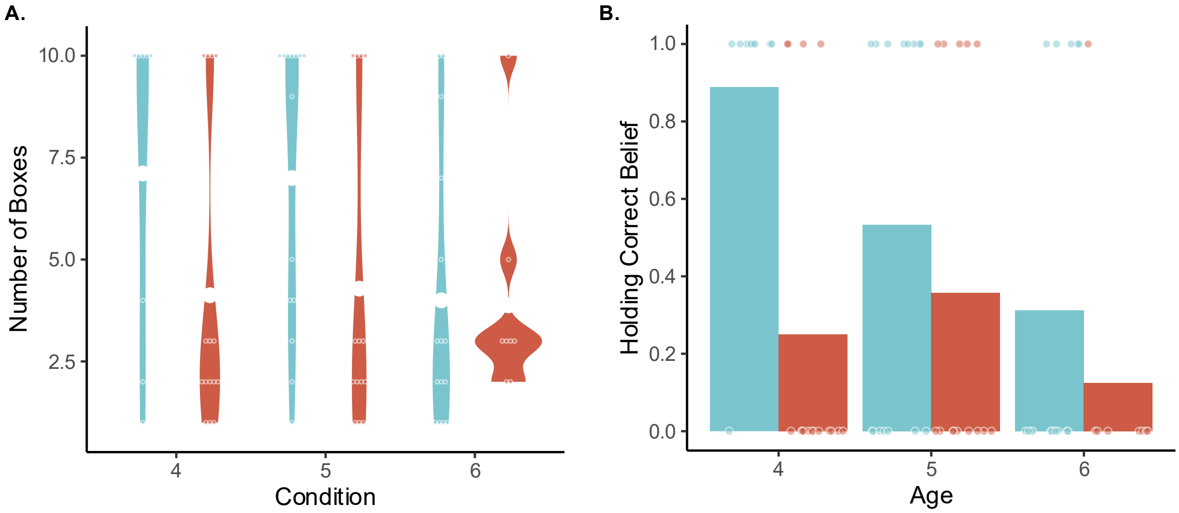

Figure S1. **Group influence on children's belief formation across age group. A.** Number of boxes children opened by condition and age group (n = 78 biologically independent replicates (children)). The distribution of responses for children in the No Group Condition is shown in blue, and for children in the Group Condition in red. Individual data points (open circles) and condition means (closed circles) are plotted in white. **B.** Proportion of children correctly believing there were more lions in the boxes by condition and age group in Study 1 (n = 78 biologically independent replicates). Blue bars represent children in the No Group Condition and red bars represent children in the Group Condition. Colored circles represent individual data points for each condition by age group.

To check how much participants liked a group member from each group and how participants divided five stickers between them, we used linear models. For both models, the difference-score was a numeric response variable (coded as the difference between the two groups; 0-5). The full models included the predictor condition (Group, No Group). We checked the model residuals visually, which fulfilled the assumption to be normally distributed. We also checked for influential cases by comparing model coefficients between a model based on all data and model coefficients based on data with cases excluded one at a time. No influential cases were identified. Homoscedasticity was assessed using the Breusch–Pagan test (p = .42) from the lmtest package (version 0.9.40) and visual inspection of the residuals versus fitted values plot. To confirm the validity of our results, we additionally report a non-parametric Mann–Whitney U test.

We then compared the model fit of the full models to the model fit of the respective null models without condition. There was a marginal trend towards an effect of condition for liking-ratings ($\chi^2(1) = 14.17$, $p = .055$, $R^2 = 0.05$). In the Group Condition, children liked their group an average of 3.8 units (SD = 1.2) and the other group an average of 2.6 units (SD = 1.7), while children in the No Group Condition liked the first group an average of 3.5 units (SD = 1.2) and the second group an average of 3.2 units (SD = 1.58). An additional Mann-Whitney U Test indicated an effect of condition ($W = 416$, $p = .027$).

There was a significant effect of condition for sticker-divisions ($\chi^2(1) = 48.29$, $p < .001$, $R^2 = 0.19$). Children in the Group Condition gave more stickers to their ingroup (mean = 3.6, SD = 1.0) over the outgroup (mean = 1.2, SD = .8), than children in the No Group Condition gave stickers to group 1 (mean = 2.7, SD = 1.1) over group 2 (mean = 2.0, SD = .8). The effect was confirmed with a Mann-Whitney U Test ($W = 292$, $p < .001$).

Table S4. **Full model (lm) output for the liking ratings as well as sticker allocation in Study 1**. Estimates, standard errors (SE), and likelihood ratio test output for the single effects. The factor condition was dummy coded with the No Group Condition as the reference level. Confidence intervals were derived using the confint function. Likelihood ratio tests used χ^2 statistics and were two-sided. To account for multiple comparisons, we first conducted a full-null model comparison before testing individual effects.

	estimates	SE	CI_{Lower}	CI_{Upper}	χ^2	*df*	*p*
Liking							
(Intercept)	0.31	0.33	-0.35	0.96			
Condition	0.90	0.47	-0.03	1.84	14.17	1	.055

Sticker							
(Intercept)	0.75	0.29	0.18	1.32			
Condition	1.66	0.41	0.84	2.48	48.29	1	<.001

Study 2: Description of the statistical analysis

To assess children's confidence following exposure to evidence, we fitted a linear model predicting children's confidence (1 – 9, continuous numeric) by condition (Group, No Group, factor). We checked the model for influential cases, which was no problem. We then checked the model residuals visually. Assumption of normality and homoscedasticity were fulfilled, as determined with a Breusch–Pagan test ($p = 0.5$) and visual inspection of the residuals versus fitted values plot. The full model containing condition (Group, No Group) was a significantly better fit than the null model ($\chi^2(1) = 128.03$, $p < .001$, $R^2 = 0.11$). Thus, as predicted, children in the Group Condition showed stronger confidence after seeing evidence than children in the No Group Condition. Children in the No Group Condition possessed an average confidence of 5.13 units (SD = 3.16), while children in the Group Condition possessed an average confidence of 7.16 units (SD = 2.62). These results were confirmed by an additional non-parametric test ($W = 1224$, $p < .001$).

Full Model for Confidence	Confidence (1-9) ~ Condition (No Group / Group Condition)

In an additional exploratory analysis, we fitted a linear model predicting confidence by condition (Group, No Group, factor), age (4-6, continuous numeric), and their interaction (like our full models in Study 1). Here, the normality and homoscedasticity assumption were not fully met. While the Breusch–Pagan test was not significant, the residual plot indicated a pattern. To address this inconsistency, we confirmed the validity with of our findings with an additional GLMM reported below. The full model was significantly different compared to a null model containing only the intercept ($\chi^2(3) = 133.22$, $p < .001$, $R^2 = 0.11$). Neither the interaction effect between condition and age ($\chi^2(3) = 1.15$, $p = .709$, $R^2 < 0.01$), nor the main effect of age were statistically significant ($\chi^2(1) = 4.03$, $p = .485$, $R^2 < 0.01$). However, the condition effect remained significant even when controlling for age ($\chi^2(1) = 125.73$, $p < .001$, $R^2 = 0.11$).

Table S5. **Full pre-registered and exploratory model (lm) output for confidence in belief in Study 2**. Estimates, standard errors (SE), and likelihood ratio test output for the single effects. The factor condition was dummy coded

with the No Group Condition as the reference level. Confidence intervals were derived using the confint function. Likelihood ratio tests used χ^2 statistics and were two-sided. To account for multiple comparisons, we first conducted a full-null model comparison before testing individual effects.

	estimates	SE	CI_{Lower}	CI_{Upper}	χ^2	*df*	*p*
Pre-registered model							
(Intercept)	5.13	0.37	4.4	5.86			
Condition	2.03	0.52	1.0	3.07	128.03	1	< .001
Exploratory model							
(Intercept)	3.39	2.39	-1.33	8.12			
Condition	3.20	3.28	-3.28	9.69	125.73	1	<.001
Age	0.31	0.43	-0.53	1.16	4.03	1	.485
Condition: Age	-0.21	0.58	-1.37	0.94	1.15	1	.709

To ensure the validity of our results, we also fitted an additional GLMM. In this model, each point on the confidence scale was treated as a binary response variable (yes/no), with each participant having the possibility of choosing nine points in total. We included the predictors condition, age, and their interaction as fixed effects, and a random intercept for individual identity. Collinearity was not an issue and there was an absence of influential cases.

The full model for was a significantly better fit than the null model ($\chi^2(3) = 15.43$, $p = .001$, $R^2 = 0.14$). We then compared a reduced model without the interaction term to models without a predictor of interest. We found a significant effect of condition ($\chi^2(1) = 15.06$, $p < .001$, $R^2 = 0.09$), such that how many boxes children opened varied across the No Group and Group Conditions. There was no statistically significant effect of age ($\chi^2(1) = 0.02$, $p = .887$, $R^2 < 0.01$). Lastly, we compared the reduced model without the interaction term to the full model. There was no statistically significant interaction between condition and age ($\chi^2(1) = 0.27$, $p = .602$, $R^2 < 0.01$). Taken together, these results demonstrate that the effect of condition is consistent across multiple analytic approaches.

Table S6. **Additional model (GLMM) output for confidence in belief in Study 2.** Estimates, standard errors (SE), and likelihood ratio test output for the single effects. The factor condition was dummy coded with the No Group Condition as the reference level. Confidence intervals were derived using the 1000 parametric bootstraps. Likelihood ratio tests used χ^2 statistics and were two-sided. To account for multiple comparisons, we first conducted a full-null model comparison before testing individual effects.

	estimates	SE	CI_{Lower}	CI_{Upper}	χ^2	*df*	*p*

(Intercept)	-0.26	2.28	-5.08	4.36			
Condition	3.82	3.35	-3.11	11.19	15.06	1	< .001
Age	0.19	0.41	-0.63	1.07	0.02	1	.887
Condition: Age	-0.31	0.59	-1.58	0.89	0.27	1	.602

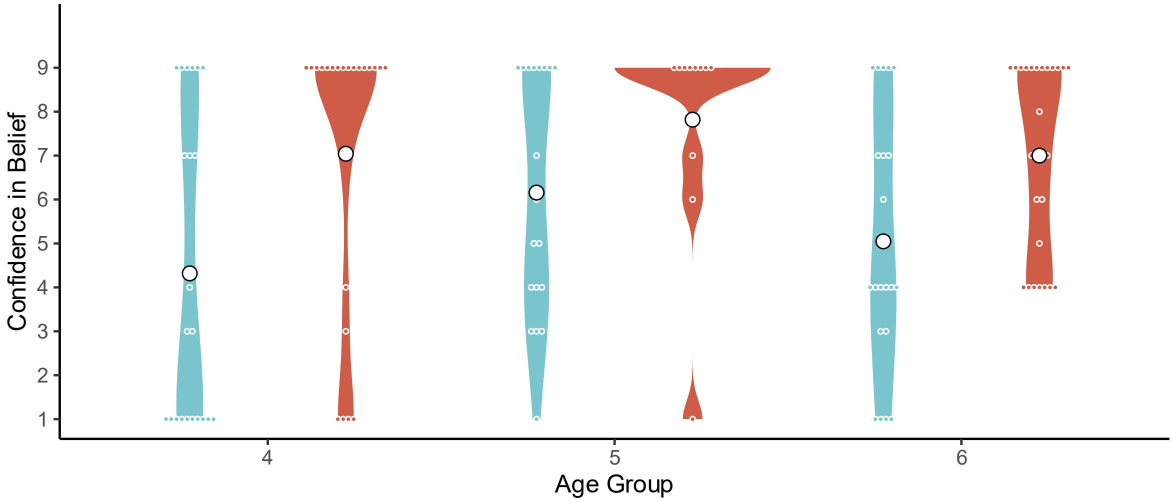

Figure S2. **Group influence on children's evaluation of group-supporting evidence in Study 2.** Children's confidence in belief after seeing evidence by condition and age group in Study 2. The distribution of responses for children in the No Group Condition is in blue and the distribution of responses for children in the Group Condition is in red. Colored circles represent individual data points, and white circles indicate the means for each condition by age group (n = 124 biologically independent replicates).

Next, we analyzed the results of the preference questions — how much participants liked a group-member from each group and how participants divided five stickers between the group members. We used linear models predicting the difference-score (difference between the ingroup and the outgroup for children in the Group Condition or group 1 and group 2 in the No Group Condition), an ordinal response variable for both liking-ratings and sticker-divisions. The full models included the predictor condition (Group, No Group). We compared the model fit of the full models to the model fit of the respective null models without condition. The assumption of homoscedasticity was fulfilled, as assessed using the Breusch–Pagan test (non-significant, liking-ratings: $p = .15$, sticker-division: $p = .67$) and residual plots.

When assessing how much children liked the two groups, we found a significant effect of condition ($\chi^2(1) = 41.07$, $p < .001$, $R^2 = 0.13$). In the Group Condition, children liked their group an average of 3.3 units (SD = 0.8) and the other group an average of 2.4 units (SD = 1.0), while children in the No Group Condition liked the first group an average of 2.7 units (SD = 1.0) and the second group an average of 3.0 units (SD = 1.0). This finding was confirmed with the non-

parametric Mann–Whitney U test (W = 999, p = .001). We did not find a statistically significant effect of condition for the sticker-divisions ($\chi^2(1)$ = 6.05, p = .176, R^2 = 0.02). Children in the Group Condition gave an average of 2.8 stickers to their ingroup (SD = 1.0) and 2.0 stickers to the outgroup (SD = 1.0), while children in the No Group Condition gave an of average 2.5 stickers to group 1 (SD = 1.1) and 2.1 stickers to the group 2 (SD = 1.0). This finding was confirmed in the non-parametric test (W = 1386, p = .064).

Table S7. **Full model (lm) output for the liking ratings as well as sticker allocation in Study 2**. Estimates, standard errors (SE), and likelihood ratio test output for the single effects. The factor condition was dummy coded with the No Group Condition as the reference level. Confidence intervals were derived using the confint function. Likelihood ratio tests used χ^2 statistics and were two-sided. To account for multiple comparisons, we first conducted a full-null model comparison before testing individual effects.

	estimates	SE	CI_{Lower}	CI_{Upper}	χ^2	*df*	*p*
Liking							
(Intercept)	-0.27	0.21	-0.68	0.14			
Condition	1.18	0.29	0.61	1.76	41.07	1	<.001
Sticker							
(Intercept)	0.37	0.24	-0.10	0.84			
Condition	0.45	0.34	-0.21	1.12	6.05	1	.176

Study 3: Description of the statistical analysis

We first looked at how confident children initially were in their beliefs when we asked them before seeing counterevidence. We fitted a linear model predicting children's initial confidence (initial distance from the midpoint, 0 – 4) by condition (Group, No Group). Since the assumption of normality of residuals was not met, we confirmed the validity of our findings with non-parametric test. For the linear model, the full model containing condition was not a significantly better fit than the null model ($\chi^2(1)$ = 0.82, p = .618, R^2 < 0.01). The Mann-Whitney U Test confirmed this finding (W = 497.5, p = 0.45).

Table S8. **Full model (lm) output for the initial confidence in the belief, Study 3**. Estimates, standard errors (SE), and likelihood ratio test output for the single effects. The factor condition was dummy coded with the No Group condition as the reference level. Confidence intervals were derived using the confint function. Likelihood ratio tests used χ^2 statistics and were two-sided. No adjudgments were made for multiple comparisons.

	estimates	SE	CI_{Lower}	CI_{Upper}	χ^2	*df*	*p*

(Intercept)	2.57	0.33	1.91	3.23			
Condition	-0.23	0.47	-1.17	0.70	0.8	1	.618

As pre–registered, to assess whether group membership affected children's evaluation of counterevidence, we fitted a linear model predicting reduction-in-confidence (calculated as a difference score between their Belief Measure 1 and 2 ratings; 0 - 8) by condition. We checked the model for normal distribution of the residuals, homoscedasticity, and absence of influential cases. All assumptions were fulfilled. Next, we compared the full model to a null model without condition as a predictor. The full model containing condition (Group, No Group) was a significantly better fit than the null model ($\chi^2(1) = 60.00$, $p = .019$, $R^2 = 0.09$), such that children showed a stronger reduction in confidence in the Group Condition. This finding was confirmed with a non-parametric Mann-Whiteny U Test ($W = 608.5$, $p = .02$).

Full Model for Difference- score	Diff-score (0-8) ~ Condition (No Group / Group Condition)

In an exploratory analysis, we also fitted a linear model predicting reduction-in-confidence by condition (Group, No Group), age (continuous, 4-6), and their interaction (like our full models in Study 1). All assumptions (normality and homoscedasticity of residuals, absence of influential cases and collinearity) were fulfilled. The full model was not a statistically significantly better fit than the null model ($\chi^2(3) = 76.44$, $p = .072$, $R^2 = 0.11$), and neither the interaction effect between age and condition ($\chi^2(1) = 15.48$, $p = .221$, $R^2 = 0.02$), nor the main effect of age were statistically significant ($\chi^2(1) = 0.97$, $p = .761$, $R^2 < 0.01$). However, the condition effect remained significant when controlling for age ($\chi^2(1) = 60.89$, $p = .018$, $R^2 = 0.09$).

Table S9. **Full pre-registered and exploratory model (lm) output for difference-score in Study 3**. Estimates, standard errors (SE), and likelihood ratio test output for the single effects. The factor condition was dummy coded with the No Group Condition as the reference level. Confidence intervals were derived using the confint function. Likelihood ratio tests used χ^2 statistics and were two-sided. To account for multiple comparisons, we first conducted a full-null model comparison before testing individual effects.

	estimates	SE	CI_{Lower}	CI_{Upper}	χ^2	*df*	*p*
Pre-registered model							
(Intercept)	4.23	0.60	3.03	5.44			

Condition	-2.00	0.85	-3.70	-0.30	60.00	1	.019
Exploratory model							
(Intercept)	6.48	3.77	-1.09	14.04			
Condition	-8.57	5.57	-19.73	2.59	60.89	1	.018
Age	-0.41	0.68	-1.78	0.96	.97	1	.761
Condition: Age	1.19	1.00	-0.81	3.18	15.48	1	.221

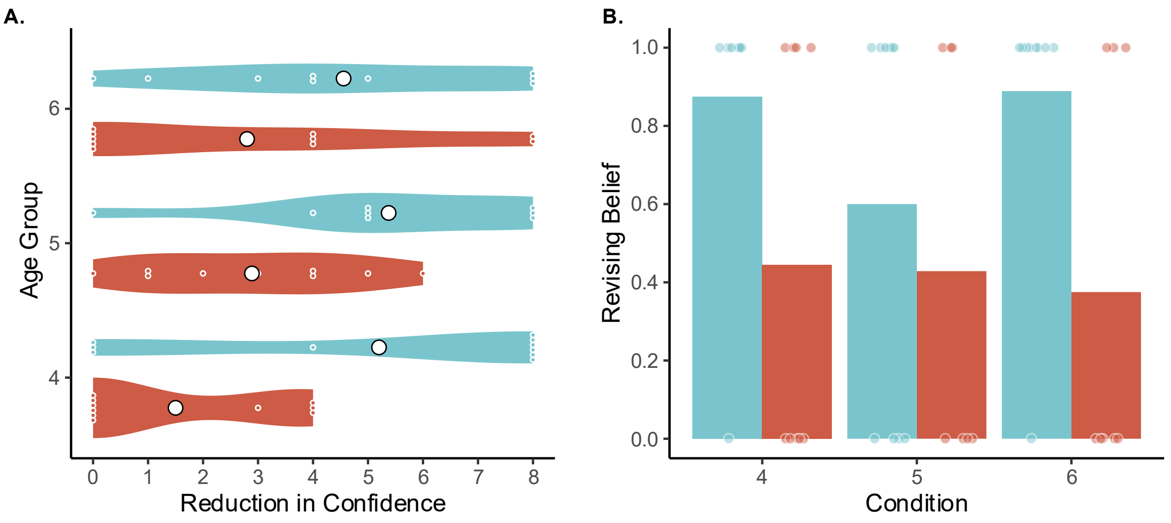

Figure S3. **Group influence on children's evaluation of group-opposing evidence in Study 3. A.** Unit reduction in confidence in children's belief after seeing counterevidence by condition and age group in Study 3. The distribution of responses for children in the No Group Condition is in blue and the distribution of responses for children in the Group Condition is in red. Colored circles represent individual data points, and white circles indicate the means for each condition by age group (n = 60 biologically independent replicates). B. Proportion of children who revised their initial beliefs (e.g., switching from believing elephant to believing in lions, or vice versa; n = 51 biologically independent replicates). Blue bars represent children in the No Group Condition and red bars represent children in the Group Condition. Colored circles represent individual data points for each condition by age group.

In an additional exploratory analysis, we assessed whether children differed across conditions in how often they revised their initial belief (e.g., switching from believing there were more elephants to more lions, or vice versa). A change across the midpoint was coded as 1 (belief revision) and no change across the midpoint as 0 (no revision). For interpretability, children whose second rating landed at the midpoint were not included in this analysis (N = 9), though conclusions remain unchanged if these children are instead coded as having revised their beliefs. We fit a generalized linear model predicting belief revision, a binary response variable (0 = no belief revision, and 1 = belief revision) predicted by condition (Group, No Group) and age (continuous, 4-6). Collinearity was not an issue (largest vif = 1.01) and there were no influential cases.

Full Model for Revising Boxes	Revise (no/yes) ~ Condition (No Group / Group Condition) + Age (4-6, continuous)

The full model was a significantly better fit than the null model ($\chi^2(2) = 1.66$, $p = .023$, $R^2 = 0.14$). Next, we compared the full model to respective reduced models without the predictor of interest. There was a significant effect of condition ($\chi^2(1) = 7.46$, $p = .006$, $R^2 = 0.18$), such that children in the No Group Condition more often revised their beliefs. There was not a significant effect of age ($\chi^2(1) = 0.01$, $p = .910$, $R^2 < 0.01$).

Table S10: **Full model (glm) output for revising initial belief in Study 3.** Estimates, standard errors (SE), and likelihood ratio test output for the single effects. The factor condition was dummy coded with the No Group Condition as the reference level. Confidence intervals were derived using the confint function. Likelihood ratio tests used χ^2 statistics and were two-sided. To account for multiple comparisons, we first conducted a full-null model comparison before testing individual effects.

	estimates	SE	CI_{Lower}	CI_{Upper}	χ^2	*df*	*p*
(Intercept)	0.82	0.43	-0.03	1.68			
Condition	-0.36	0.13	-0.62	-0.10	7.46	1	.006
Age	-0.01	0.08	-0.16	0.14	.01	1	.910

As in Study 1 and 2, to check how much participants liked a group member from each group and how participants divided five stickers between them, we used linear models predicting difference-score (coded as the difference between the two groups). The full models included the predictor condition (Group, No Group). We compared the model fit of the full models to the model fit of the respective null models without condition. The model residuals were normally distributed and homoscedasticity was fulfilled. There were also no influential cases.

We found a significant effect of condition for liking-ratings ($\chi^2(1) = 16.96$, $p < .001$, $R^2 < 0.18$), such that children in the Group Condition liked their ingroup over the outgroup (more than children in the No Group Condition liked group 1 over group 2. In the Group Condition, children liked their group an average of 2.4 units (SD = .7) and the other group an average of 1.6 units (SD = .9), while children in the No Group Condition liked the first group an average of 1.9 units (SD = .9) and the second group an average of 2.2 units (SD = .8). This finding was confirmed with a non-parametric Mann-Whitney U Test ($W = 237.5$, $p < .001$).

There was also a significant effect of condition for the sticker-divisions ($\chi^2(1) = 22.80$, p = .010, $R^2 < 0.11$), such that children in the Group Condition gave more stickers to their ingroup over the outgroup (more than children in the No Group Condition gave stickers to group 1 over group 2). Children in the Group Condition gave more stickers to their ingroup (mean = 3.1, SD = 1.2) over the outgroup (mean = 1.6, SD = 1.0), than children in the No Group Condition gave stickers to group 1 (mean = 2.3, SD = 1.0) over group 2 (mean = 2.1, SD = .8). This finding was confirmed with a non-parametric Mann-Whitney U Test ($W = 226.5$, $p = .001$).

Table S11. **Full model (lm) output for the liking ratings as well as sticker allocation in Study 3**. Estimates, standard errors (SE), and likelihood ratio test output for the single effects. The factor condition was dummy coded with the No Group Condition as the reference level. Confidence intervals were derived using the confint function. Likelihood ratio tests used χ^2 statistics and were two-sided. To account for multiple comparisons, we first conducted a full-null model comparison before testing individual effects.

	estimates	SE	CI_{Lower}	CI_{Upper}	χ^2	*df*	*p*
Liking							
(Intercept)	-0.26	0.22	-0.70	0.19			
Condition	1.09	0.31	0.48	1.71	16.96	1	< .001*
Sticker							
(Intercept)	0.18	0.35	-0.53	0.88			
Condition	1.25	0.49	0.28	2.23	22.80	1	.010

Effect of Gender in Studies 1-3

We ran exploratory analyses with gender included as a predictor for all main analyses. We did not find any statistically significant effects of gender (Study 1 Boxes: $\chi^2(1) = 1.01$, $p = .767$, $R^2 < 0.01$; Study 1 Belief: $\chi^2(1) = 0.72$, $p = .396$, $R^2 < 0.01$. Study 2 Confidence: $\chi^2(1) = 16.21$, $p = .154$, $R^2 = 0.02$; Study 3 Difference-score: $\chi^2(1) = 1.53$, $p = .700$, $R^2 < 0.01$; Study 3 Revise: $\chi^2(1) = 0.83$, $p = .363$, $R^2 = 0.02$).

References

1. Fox, J. & Weisberg, S. *An R Companion to Applied Regression*. 2nd edn. (Sage, Thousand Oaks, 2011).
2. Baayen, R. H., Davidson, D. J., & Bates, D. M. Mixed-effects modeling with crossed random effects for subjects and items. *Journal of memory and language* **59,** 390-412 (2008).
3. Nagelkerke, N. J. D. A note on a general definition of the coefficient of determination. *Biometrika* **78**, 691–692 (1991).
4. Nakagawa, S. & Schielzeth, H. A general and simple method for obtaining R^2 from generalized linear mixed-effects models. *Methods Ecol. Evol.* **4**, 133–142 (2013).
5. Tingley, D., Yamamoto, T., Hirose, K., Keele, L. & Imai, K. Mediation: R Package for Causal Mediation Analysis. *J. Stat. Software* **59**, 1–38 (2014).
