# Supplementary material for: Group membership biases children’s evaluation of evidence
Source: Nat Commun. 2025 Dec 15;16:11245. doi: 10.1038/s41467-025-66085-0 (PMC12717042; doi:10.1038/s41467-025-66085-0)
Supplement: Supplementary file 2 — Reporting Summary [file 41467_2025_66085_MOESM2_ESM.pdf]

nature portfolio

Corresponding author(s): Joshua Confer

Last updated by author(s): Jun 21, 2025

Reporting Summary

Nature Portfolio wishes to improve the reproducibility of the work that we publish. This form provides structure for consistency and transparency in reporting. For further information on Nature Portfolio policies, see our Editorial Policies and the Editorial Policy Checklist.

Statistics

For all statistical analyses, confirm that the following items are present in the figure legend, table legend, main text, or Methods section.

n/a	Confirmed	
☐	☒	The exact sample size (*n*) for each experimental group/condition, given as a discrete number and unit of measurement
☐	☒	A statement on whether measurements were taken from distinct samples or whether the same sample was measured repeatedly
☐	☒	The statistical test(s) used AND whether they are one- or two-sided<br>*Only common tests should be described solely by name; describe more complex techniques in the Methods section.*
☐	☒	A description of all covariates tested
☐	☒	A description of any assumptions or corrections, such as tests of normality and adjustment for multiple comparisons
☐	☒	A full description of the statistical parameters including central tendency (e.g. means) or other basic estimates (e.g. regression coefficient) AND variation (e.g. standard deviation) or associated estimates of uncertainty (e.g. confidence intervals)
☐	☒	For null hypothesis testing, the test statistic (e.g. *F*, *t*, *r*) with confidence intervals, effect sizes, degrees of freedom and *P* value noted<br>*Give P values as exact values whenever suitable.*
☒	☐	For Bayesian analysis, information on the choice of priors and Markov chain Monte Carlo settings
☒	☐	For hierarchical and complex designs, identification of the appropriate level for tests and full reporting of outcomes
☐	☒	Estimates of effect sizes (e.g. Cohen's *d*, Pearson's *r*), indicating how they were calculated

Our web collection on statistics for biologists contains articles on many of the points above.

Software and code

Policy information about availability of computer code

Data collection	NA
Data analysis	R version 4.5.1; packages used: tidyverse (2.0.0), lme4 (1.1.37), car (3.1.3), pwr (1.3.0), rsq (2.7), partR2 (0.9.2), MuMIn (1.48.11), mediation (4.5.1), lmtest (0.9.40), dplyr (1.1.4), svglite (2.2.1), ggbeeswarm (0.7.2).

For manuscripts utilizing custom algorithms or software that are central to the research but not yet described in published literature, software must be made available to editors and reviewers. We strongly encourage code deposition in a community repository (e.g. GitHub). See the Nature Portfolio guidelines for submitting code & software for further information.

Data

Policy information about availability of data

All manuscripts must include a data availability statement. This statement should provide the following information, where applicable:

- Accession codes, unique identifiers, or web links for publicly available datasets
- A description of any restrictions on data availability
- For clinical datasets or third party data, please ensure that the statement adheres to our policy

The anonymized raw data used in the analyses have been deposited in the Open Science Framework (OSF) project link at https://osf.io/vx7dn. All figures and tables in the main text and Supplementary Information can be reproduced using the shared data and code.

Research involving human participants, their data, or biological material

Policy information about studies with human participants or human data. See also policy information about sex, gender (identity/presentation), and sexual orientation and race, ethnicity and racism.

Reporting on sex and gender	Gender was not considered in the study design (given previous research). Information about the gender of participants was indicated by the parents of children. Study 1: 44 girls, 34 boys. Study 2: 51 girls, 73 boys. Study 3: 26 girls, 34 boys.
Reporting on race, ethnicity, or other socially relevant groupings	Information about the race/ethnicity of participants was indicated by the parents of children. Parents were provided with a drop down menu in which they could select one or more options: Hispanic or Latino, African or African American, American Indian or Native Alaskan, Asian, Pacific Islander, White, or Other. Study 1: White (40%), Asian (27%), Hispanic or Latino (9%), multiple races (9%), and other/unknown (15%). Study 2: White (29.8%), Asian (23.4%), Hispanic or Latino (6.5%), African or African American (4.0%), multiple races (16.1%), and other/unknown (20.2%). Study 3: White (33.3%), Asian (18.3%), Hispanic or Latino (20%), African or African American (1.7%), multiple races (18.3%), and other/unknown (8%).
Population characteristics	4-6-year-olds. Study 1: M = 5.53 years, SD = .82. Study 2: M = 5.56 years, SD = .90. Study 3: M = 5.52 years , SD = .87.
Recruitment	Children were recruited at local museums and zoos around the greater San Francisco Bay Area.
Ethics oversight	Committee for the Protection of Human Subjects at the University of California, Berkeley (CPHS Protocol Number: 2019-10-12605).

Note that full information on the approval of the study protocol must also be provided in the manuscript.

Field-specific reporting

Please select the one below that is the best fit for your research. If you are not sure, read the appropriate sections before making your selection.

☐ Life sciences ☒ Behavioural & social sciences ☐ Ecological, evolutionary & environmental sciences

For a reference copy of the document with all sections, see nature.com/documents/nr-reporting-summary-flat.pdf

Behavioural & social sciences study design

All studies must disclose on these points even when the disclosure is negative.

Study description	Studies 1–3 employed a quantitative design, using experimental tasks.
Research sample	We recruited 4-6-year-olds from around the greater San Francisco Bay Area. Study 1: M = 5.53 years, SD = .82, 44 girls. Study 2: M = 5.56 years, SD = .90, 51 girls. Study 3: M = 5.52 years , SD = .87, 26 girls. The sample is representative, and aligns with previous research on young children's epistemic practices.
Sampling strategy	In Study 1, we aimed to include 80 participants in our final sample following a power analysis, which suggested we would find a significant effect of condition with an average power of $1-\beta > 0.82$. In Study 2, we aimed to include 124 participants in our final sample, determined by a power analysis based on pilot data from 27 children. In the pilot, children were randomly assigned to either the Group Condition or No Group Condition, and we measured their confidence ratings after seeing evidence. The Group Condition had a mean confidence rating of 7.31 (SD = 2.56), while the No Group Condition had a mean of 5.85 (SD = 3.29). We fit a linear model predicting confidence from condition, which yielded $R^2 = .11$, corresponding to Cohen's $f^2 = 0.13$. We used this effect size in a power analysis via pwr.f2.test() from the pwr package, which indicated that 124 participants would be sufficient for 80% power at $\alpha = 0.05$. To reach our sample size, we tested 139 children. In Study 3, we aimed to include 60 participants in our final sample, determined by a power analysis based on pilot data from 27 children. In the pilot, children were randomly assigned to either the Group Condition or No Group Condition, and we measured their difference scores after seeing counterevidence. The Group Condition (M = 3.57, SD = 3.76) showed less confidence in the counterevidence than those in the No Group Condition (M = 6.15, SD = 2.73). This yielded a Cohen's d of 0.79, which we used for our power analysis via pwr.t.test() from the pwr package. This analysis indicated that a total sample size of 27 participants per group would be sufficient for 80% power at $\alpha = 0.05$, which we conservatively rounded to 30 participants per group in our pre-registration.
Data collection	Data was collected using a computer and video recording devices. The experimenters were not blind to the experimental conditions.
Timing	Study 1: October 2022 - February 2023. Study 2: September 2024 - December 2024. Study 3: June 2023 - July 2023
Data exclusions	Study 1: We excluded 13 children from our final analyses: twelve due to experimenter error and one due to parental interference, following our pre-registered criteria. Study 2: In line with our pre-registered exclusion criteria, we excluded 15 children from our final analyses: two children due to experimenter error, seven children for failure to understand the confidence scale, one child who was not able to follow along with the procedure, and five children because we had already reached our predetermined sample size. Study 3: In line with our pre-registered exclusion criteria, we excluded 15 children from our final analyses: five children due to experimenter error, five children for failure to understand the confidence scale, four children in the Group Condition who did not initially believe their group (as we were interested in how children evaluate counterevidence that went against an in-group belief), and one last child because we had already reached our predetermined sample size.

Non-participation	Three participants dropped out in Study 2 (two were non-responsive, one did not speak English)
Randomization	Participants were randomly assigned to one of the two experimental conditions.

Reporting for specific materials, systems and methods

We require information from authors about some types of materials, experimental systems and methods used in many studies. Here, indicate whether each material, system or method listed is relevant to your study. If you are not sure if a list item applies to your research, read the appropriate section before selecting a response.

Materials & experimental systems

n/a	Involved in the study
☒	☐ Antibodies
☒	☐ Eukaryotic cell lines
☒	☐ Palaeontology and archaeology
☒	☐ Animals and other organisms
☒	☐ Clinical data
☒	☐ Dual use research of concern
☒	☐ Plants

Methods

n/a	Involved in the study
☒	☐ ChIP-seq
☒	☐ Flow cytometry
☒	☐ MRI-based neuroimaging

Plants

Seed stocks	NA
Novel plant genotypes	NA
Authentication	NA
